# *Zymomonas mobilis* as a model system for production of biofuels and biochemicals

**DOI:** 10.1111/1751-7915.12408

**Published:** 2016-09-15

**Authors:** Shihui Yang, Qiang Fei, Yaoping Zhang, Lydia M. Contreras, Sagar M. Utturkar, Steven D. Brown, Michael E. Himmel, Min Zhang

**Affiliations:** ^1^ National Bioenergy CenterNational Renewable Energy LaboratoryGoldenCO80401USA; ^2^ Hubei Collaborative Innovation Center for Green Transformation of Bio‐resourcesHubei Key Laboratory of Industrial BiotechnologyCollege of Life SciencesHubei UniversityWuhan430062China; ^3^ School of Chemical Engineering and TechnologyXi'an Jiaotong UniversityXi'an710049China; ^4^ Great Lakes Bioenergy Research CenterWisconsin Energy InstituteUniversity of WisconsinMadisonWI53726USA; ^5^ McKetta Department of Chemical EngineeringUniversity of TexasAustinTX78712USA; ^6^ Graduate School of Genome Science and TechnologyUniversity of TennesseeKnoxvilleTN37919USA; ^7^ BioEnergy Science CenterOak Ridge National LaboratoryOak RidgeTN37831USA; ^8^ Biosciences DivisionOak Ridge National LaboratoryOak RidgeTN37831USA; ^9^ Biosciences CenterNational Renewable Energy LaboratoryGoldenCO80401USA

## Abstract

*Zymomonas mobilis* is a natural ethanologen with many desirable industrial biocatalyst characteristics. In this review, we will discuss work to develop *Z. mobilis* as a model system for biofuel production from the perspectives of substrate utilization, development for industrial robustness, potential product spectrum, strain evaluation and fermentation strategies. This review also encompasses perspectives related to classical genetic tools and emerging technologies in this context.

## Introduction

Replacement of petroleum with lignocellulosic biofuels is critical for environmental protection, energy independence and a sustainable economy. Various types of advanced biofuels under development today have high energy density and are compatible with current fuel infrastructure, including higher alcohol‐based fuels, hydrocarbon‐based fuels and fatty acid‐based fuels (Atsumi *et al*., 2008; Connor and Liao, 2009; Peralta‐Yahya and Keasling, 2010; Peralta‐Yahya *et al*., 2011). Many microorganisms are being developed for biofuel production, but all have certain limitations as economical production strains, such as industrial robustness, substrate utilization, productivity and yield. Yeast strains are among the current leading industrial biocatalyst microorganisms for fuel production (Hahn‐Hagerdal *et al*., 2006). However, engineered bacteria such as *Escherichia coli*,* Zymomonas mobilis*,* Corynebacterium glutamicum* and *Bacillus subtilis* are being developed and deployed to address commercially important biocatalyst requirements (Dien *et al*., 2003; Alper and Stephanopoulos, 2009; Smith *et al*., 2010; Blombach and Eikmanns, 2011).

*Z. mobilis* is a natural ethanologen and has many desirable industrial biocatalyst characteristics, such as high‐specific productivity, high alcohol tolerance, a broad pH range for production (pH 3.5–7.5), and the generally regarded as safe status (Swings and De Ley, 1977; Rogers *et al*., 1984, 2007; Gunasekaran and Raj, 1999; Dien *et al*., 2003; Panesar *et al*., 2006). Compared with the classical model ethanologen, *Saccharomyces cerevisiae*, which uses the Embden‐Meyerhof‐Parnas (EMP) pathway for glycolysis, *Z. mobilis* uses the Entner‐Doudoroff (ED) pathway. The ED pathway is found in strict aerobic microorganisms and conducts fermentation with 50% less ATP produced relative to the EMP pathway, which leads to improved ethanol yield. Moreover, *Z. mobilis* has a high‐specific cell surface area and consumes glucose faster than *S. cerevisiae*, leading to higher ethanol productivity than *S. cerevisiae* (Conway, 1992). Furthermore, *Z. mobilis* is a facultative anaerobic microorganism, which reduces the production cost for advanced aeration control during fermentation process scale‐up. The possibility to substitute freshwater with seawater in the culture medium could further mitigate the socio‐environmental challenges for the expansion of ethanol production (Swings and De Ley, 1977; Goncalves *et al*., 2015). In addition to the ongoing efforts to engineer *Z. mobilis* for fermentation under heat stress conditions without supplementation of amino acids and vitamins (Jia *et al*., 2013; Zhang *et al*., 2013a; Wang *et al*., 2016), a recent report demonstrated that *Z. mobilis* can utilize N_2_ as a nitrogen source and thus replace NH_4_ or the industrial nitrogen supplement, corn steep liquor. It was also observed that nitrogen fixation did not affect ethanol yield, but rather increased the specific ethanol productivity at lower biomass loadings, which could significantly reduce the cellulosic ethanol production cost by millions of dollars annually (Kremer *et al*., 2015), although the utility of this process at an industrial scale requires further investigation.

Quite a few excellent reviews are available on the ecology, physiology and historical milestones of *Z. mobilis* development (Doelle *et al*., 1993; Kalnenieks, 2006; Panesar *et al*., 2006; Rogers *et al*., 2007; He *et al*., 2014; Weir, 2016). In this review, we will briefly discuss work to develop *Z. mobilis* as a model system for biofuel and biochemical production from the perspectives of substrate utilization, robustness development, potential product spectrum, strain evaluation and fermentation strategies; as well as classical genetic tools and emerging technologies.

## Substrate utilization

### Pure sugar utilization

Wild‐type *Z. mobilis* was isolated primarily from alcoholic liquids in natural environments containing fermentable sugars, such as plant saps, and can only utilize a limited carbon source, including glucose, fructose and sucrose (Weir, 2016). To develop *Z. mobilis* as an effective production strain to utilize both C6 and C5 (especially xylose) sugars from pretreated lignocellulosic biomass, various approaches, including metabolic engineering and lab‐directed evolution, have been used. The first recombinant xylose utilization *Z. mobilis* strain was reported in 1995 and was achieved by engineering the *xylA/B* operon, *tal* and *tkt* genes from *E. coli* into *Z. mobilis* (Zhang *et al*., 1995). Since then, recombinant and evolved *Z. mobilis* strains for xylose and arabinose utilization have been developed through metabolic engineering and adaptation or similar methods. Importantly, some recombinant strains of *Z. mobilis* have been improved to utilize glucose, xylose and arabinose derived from lignocellulosic feedstock simultaneously for the fermentation of bioethanol (Chou *et al*., 2015; Deanda *et al*., 1996; Mohagheghi *et al*., 2002, 2014; Jeon *et al*., 2005; Agrawal *et al*., 2011; Ma *et al*., 2012; Yanase *et al*., 2012; Zhang *et al*., 2013a; Dunn and Rao, 2014; Wang *et al*., 2016).

### Biomass feedstocks

Many current fermentation‐based bioprocesses still rely on starch‐based carbon sources derived from food (e.g. grain and corn) (OECD‐FAO, 2015). Therefore, much effort has focused on exploring new alternative carbon sources, such as lignocellulosic biomass. Besides corn stover that has been used extensively as lignocellulosic biomass, diverse materials including energy crops have been used for ethanol production by *Z. mobilis* (Zhang and Lynd, 2010; Behera *et al*., 2012; He *et al*., 2013; Saharkhiz *et al*., 2013; Yang *et al*., 2013; Zhang *et al*., 2013b; Peralta‐Contreras *et al*., 2014; Todhanakasem *et al*., 2014; Gu *et al*., 2015; Ma *et al*., 2015; Serate *et al*., 2015; Schell *et al*., 2016a; Sulfahri *et al*., 2016); these include energy crops (sugarcane, sugar beet, carob, sweet potato and sweet sorghum), energy plants (e.g. switchgrass), industrial wastes (soybean meal, a co‐product of the production of soybean oil and maize meals), food waste, agricultural residues (corncob residues, rice bran, sweet sorghum stalk, sugarcane molasses, bamboo residues and waste paper sludge), as well as algal biomass from *Spirogyra hyalina*. This broad range of carbon sources that *Z. mobilis* can utilize, especially those from industrial, agricultural and municipal waste, will help transform waste materials into valuable biofuels or chemicals, and facilitate its commercial application in varied locations.

### Consolidated bioprocessing candidate development

Instead of relying on pretreatment and separate enzymatic hydrolysis processes to release the monosaccharides from lignocellulosic feedstocks, consolidated bioprocessing (CBP) presents a promising technology for cost‐competitive biofuel production by combining cellulase production, lignocellulose hydrolysis and sugar fermentation into a single step. In general, two different strategies have been pursued to engineer CBP strains: (i) a naturally cellulolytic microorganism (e.g. *Clostridium thermocellum*) could be improved metabolically for economic biofuels production; or (ii) a biofuel producing strain (e.g. *Saccharomyces cerevisiae*) could be improved to utilize lignocellulose by the heterologous expression of fungal cellulases (Lynd *et al*., 2005; Olson *et al*., 2012).

Genomic information reveals the existence of an endoglucanase (EC3.2.1.4) homologue in *Z. mobilis* (ZMO1086) that has been characterized previously (Rajnish *et al*., 2008). However, the critical cellobiohydrolase (EC3.2.1.91) and β‐d‐glucosidase (EC3.2.1.21) cellulase families are not represented in *Z. mobilis*. To achieve the goal of cellulosic biomass degradation capability, cellulases from other species have been expressed in *Z. mobilis* and enzyme activities have been detected, such as the ‘carboxymethylcellulase’ from *Cellulomonas uda* CB4 and *Acetobacter xylinum* IFO 3288 (Misawa *et al*., 1988; Okamoto *et al*., 1994), two cellulolytic endo‐1,4‐β‐d‐glucanase (EC3.2.1.4) enzymes (GH5 and GH12) from *Acidothermus cellulolyticus* (Linger *et al*., 2010), and an endo‐1,4‐β‐d‐glucanase from *Enterobacter cloacae* (Vasan *et al*., 2011). Recently, a heterologous β‐d‐glucosidase from *Bacillus polymyxa* was expressed in *Z. mobilis*, where the signal peptide ZMO1086 facilitated its secretion (Luo and Bao, 2015). A review article summarizing cellulase gene expression in *Z. mobilis* can be consulted for more information (Jung *et al*., 2012).

Although these results suggest that *Z. mobilis* can express cellulases and thus has the potential to be engineered to be an effective CBP strain, enzyme optimization as well as the selection of an appropriate promoter, signal peptide and secretion pathway need to be considered. For example, because cellulase synthesis and secretion could be energetically costly for the cells when exoenzymes are expressed and secreted, it would be ideal if expression could be turned off once biomass hydrolysis is complete to reserve cellular energy for biofuel production. In addition, there is a ‘chicken and egg’ problem here for CBP strategy – cells must produce and secret cellulases extracellularly to degrade lignocellulose and also supply carbon and energy for normal cellular growth; however, they must first grow to initiate transcription and translation for cellulase production (Kricka *et al*., 2014). Furthermore, the abundant production of multiple heterologous enzymes can reduce cell fitness for phenotypes like biofuel tolerance and growth rate. This may in part explain challenges (e.g. long fermentation lag time and low productivity) associated with CBP candidates for high titre biofuel production using lignocellulosic biomass substrates. Despite the promising role that CBP may eventually play in commercial biofuels production, metabolic engineering efforts for ethanologens support more focused work on engineering *Z. mobilis* for advanced fuels production when paired with the advanced cellulase preparations available today.

## Inhibitors and microbial robustness development

Microorganisms are subjected to various stresses, including the perturbing environmental factors of temperature, pH and oxygen; as well as toxic compounds from growth substrates, metabolic intermediate and fermentation products. For example, toxic hydrolysate inhibitors are considered a key barrier for value‐added chemical production from biomass and microbial biocatalyst robustness is an important parameter to develop microbial strains for industrial applications (Winkler and Kao, 2014). Although *Z. mobilis* is very tolerant to its end‐product, ethanol and to many individual inhibitors derived from biomass deconstruction and hydrolysis, synergistic effects among these compounds still have detrimental effects on cell growth and ethanol production of *Z. mobilis*.

### Pretreatment and hydrolysate inhibitors

The purpose of pretreatment is to partially deconstruct biomass (plant cell walls) with lignin and other residuals removed in some cases, so that enzymatic hydrolysis of cellulose and hemicellulose can be achieved more rapidly with greater yields. The potential sugar streams from biomass pretreatment of hardwoods and grasses includes the monosaccharides: glucose, xylose, arabinose; as well as a host of minor compounds which depend on the chemical (Harmsen *et al*., 2010; Mood *et al*., 2013), physical (Mosier *et al*., 2005; Harmsen *et al*., 2010), physico‐chemical (Sun and Cheng, 2002; Wyman *et al*., 2005) and biological (Wyman *et al*., 2005; Sindhu *et al*., 2016) pretreatment used. The focus on pretreatment and conditioning research improves the digestibility (such as sugar consumption rates), solid concentrations and ethanol production from lignocellulosic feedstocks (Esteghlalian *et al*., 1997; Mohagheghi *et al*., 2004; Mosier *et al*., 2005; Kumar *et al*., 2009). The physico‐chemical pretreatment approach using ammonia fibre expansion (AFEX) is carried out using liquid ammonia combined with the steam explosion process (high temperature and pressure) (Bals *et al*., 2011). AFEX is reported to provide a higher sugar recovery efficiency and lower sugar loss and inhibitor formation compared with diluted acid (DA) pretreatment (Mathew *et al*., 2016). However, the high energy requirement and the use of ammonia in AFEX may increase the pretreatment cost (Mood *et al*., 2013). DA pretreatment is one of the most cost effective methods reported and has been extensively studied (Harmsen *et al*., 2010). However, inhibitory compounds formed during this process, including furfural, hydroxymethylfurfural (HMF), formic acid, levulinic acids, acetic acid, vanillin and phenolic aldehydes, have negative effects on cellular growth, metabolism and the production of desired products (Delgenes *et al*., 1996; Gu *et al*., 2015; Yi *et al*., 2015; Park *et al*., 2016). Detailed work has been conducted to investigate the composition of hydrolysate to find inhibitory compounds, and a high‐throughput biological growth assay has been developed to obtain detailed inhibitory kinetics for individual compounds or synergistic combinations of these compounds (Franden *et al*., 2009, 2013; Wang *et al*., 2014; Yi *et al*., 2015).

### Strategies to overcome toxic compounds

To overcome the impact of toxic end‐products and inhibitory compounds released from pretreatment and enzymatic hydrolysis, intensive research programs have been supported internationally. First, efforts have focused on understanding the toxic compounds in the hydrolysate as well as the effect of these toxic compounds on various host microorganisms. It was found that acetate, furfural and phenolic aldehydes are the major identifiable inhibitory compounds in the hydrolysate of pretreated biomass for *Z. mobilis* (Franden *et al*., 2009, 2013; Wang *et al*., 2014; Gu *et al*., 2015; Yi *et al*., 2015). However, phenolic acids (such as ferulic acid and *p*‐coumaric acid) and their amides are the most abundant inhibitor in AFEX‐pretreated corn stover and switchgrass (Keating *et al*., 2014; Serate *et al*., 2015).

Subsequently, the strategies by exploring novel methodologies to alleviate hydrolysate toxicity by reducing the severity of pretreatment were developed, including deacetylation and disc refining, and deacetylation and mechanical refining (DMR). These methods not only greatly improved the digestibility, sugar consumption rates, and decrease in solids concentration of lignocellulosic biomass, but also reduced the concentration of inhibitory compounds, such as the major inhibitor of acetate in the hydrolysate, resulting in high ethanol production (Esteghlalian *et al*., 1997; Mosier *et al*., 2005; Kumar *et al*., 2009; Chen *et al*., 2015, 2016). For example, the DMR process applied to corn stover achieved high sugar concentrations (230 g l^−1^) and low chemical inhibitor concentrations, which allowed fermentation to ethanol with titres as high as 86 g l^−1^ without hydrolysate detoxification and/or concentration (Chen *et al*., 2016).

In addition, genetic approaches, including forward and reverse genetics, were applied to enhance the robustness of *Z. mobilis*. The forward genetics approaches use mutagenesis (e.g. chemical mutagen or transposon‐based mutagenesis) and lab‐directed evolution to generate and select mutants of the desired phenotype. Reverse genetics is an omics‐guided metabolic engineering effort used to confirm the association of differentially expressed genetic candidates with desired phenotypes and then to transfer genetic candidates into the target host for robustness improvement. The forward genetics approaches of conventional mutagenesis, transposon mutagenesis and lab‐directed evolution have been previously reviewed (Panesar *et al*., 2006; He *et al*., 2014). Several recent successful examples include the enhancement of *Z. mobilis* stability towards known hydrolysate inhibitors (e.g. acetate, furfural) or the complex hydrolysate itself using lab‐directed evolution (Mohagheghi *et al*., 2015; Shui *et al*., 2015). Recent examples to successfully increase inhibitor tolerance through reverse genetics include the identification of Hfq using microarray studies and the demonstration of its role in conveying tolerance to multiple hydrolysate inhibitors, such as acetate, vanillin, furfural and HMF (Yang *et al*., 2009a, 2010a). Also, noteworthy is the identification of several phenolic aldehyde responsive reductase encoding genes (ZMO1116, ZMO1696 and ZMO1885) and the increased tolerance against phenolic aldehyde inhibitors, especially 4‐hydroxybenzaldehyde and vanillin, by overexpressing these genes (Yi *et al*., 2015).

Nevertheless, the forward and reverse genetic engineering strategies are closely connected. For instance, the genetic elements and mechanism of inhibitor tolerance for mutants generated through forward genetics approaches with desired phenotype can be deciphered using reverse genetics approaches (Yang *et al*., 2010b, 2015). Also, mutants generated from reverse genetics studies can be further improved using forward genetics approaches.

## Bioproducts

### Bioethanol commercial production

The most established product by *Z. mobilis* recombinant strains is ethanol, which has been extensively investigated. In addition, ethanol production genes (*pdc* and *adh*) have been utilized in various other microorganisms for ethanol production, including *E. coli* (Piriya *et al*., 2012). In contrast to the bioethanol derived from food‐based sugars, cellulosic bioethanol produced from lignocellulosic materials can be more economic and sustainable. Based on a techno‐economic analysis (TEA) model released by the National Renewable Energy Laboratory (NREL), the minimum selling price for lignocellulose‐based bioethanol can be as low as $2.15/gal gasoline equivalent (GGE) from a ‘n^th^‐plant’ conceptual design (Humbird *et al*., 2011). The entire process design included feed handling, pretreatment and conditioning, enzymatic hydrolysis and fermentation, cellulose enzyme production, product recovery, wastewater treatment, storage, onsite combustion and utilities. The process described in this TEA report used corn stover as the feedstock, which was pretreated prior to fermentation of the resulting glucose and xylose for the production of ethanol by *Z. mobilis*. The *Z. mobilis* recombinant strain was then evaluated at the pilot plant scale with other ethanologens, including recombinant yeast strains provided by commercial bioethanol producers.

Recent progress since the accomplishment of the $2.15/GGE cellulosic bioethanol goal could further drive the price down. For example, a high overall bioethanol yield of 80% and bioethanol titres of more than 60 g l^−1^ were achieved in a cultivation of *Z. mobilis* using lignocellulosic hydrolysate as the sole carbon source (Schell *et al*., 2016b). Also, a recombinant *Z. mobilis* strain utilizing hydrolysate from the DMR process produced bioethanol with titres as high as 86 g l^−1^, as discussed above (Chen *et al*., 2016). Recently, a recombinant *Z. mobilis* strain was developed to incorporate multiple gene modules, including the *xylA/xlyB/tktA/talB* operon for xylose utilization, the *metB/yfdZ* operon for lysine and methionine biosynthesis, the thioesterase gene *tesA* to enhance free fatty acid biosynthesis for increasing ethanol tolerance, a proton‐buffering peptide operon for acid stress tolerance, and a small heat shock protein operon for heat stress tolerance (Wang *et al*., 2016). The final recombinant strain TMY‐HFPX can produce ethanol up to 136 g l^−1^ from 295 g l^−1^ glucose in very high gravity (VHG) fermentation conditions without the supplementation of exogenous amino acids and vitamins with a theoretical yield of 90% (Wang *et al*., 2016).

In addition, research efforts and investments are being made to accelerate development of cellulosic bioethanol at commercial scale. Recently, the recombinant strain developed by DuPont and NREL will be utilized for a commercial‐scale bioethanol biorefinery in Nevada, IA. This project will become the world's largest commercial‐scale lignocellulose‐based bioethanol refinery opened to date, which will produce 30 million gallons of bioethanol per year. In addition, DuPont has signed agreements with Macedonian and Chinese partners to license this cellulosic bioethanol technology, including plans to produce 1.7 billion gallons of bioethanol by 2020 in Liaoning, China. Besides fulfilling energy security and helping economic recovery as an energy form and fuel additive of oxygenate, cellulosic bioethanol is cleaner than the alternative, with 90% fewer greenhouse gas emissions than gasoline (Wang *et al*., 2012).

### Native and heterologous products

**Figure 1:**
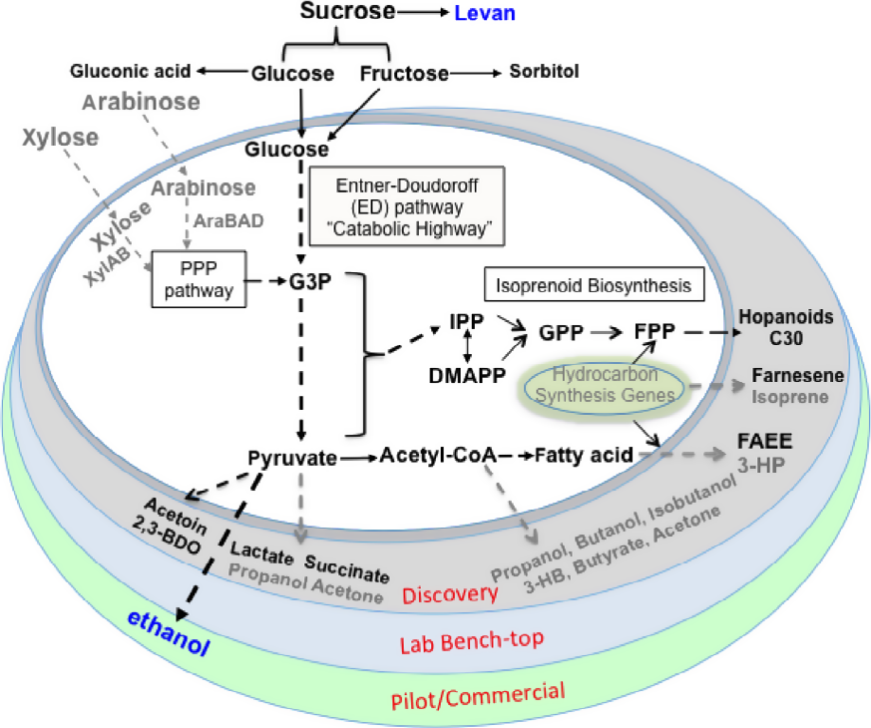
From substrate utilization to bioproducts of native and heterologous products using *Z. mobilis* as the microbial biocatalyst. Discovery, Lab Bench‐top and Pilot/Commercial scale indicate different commercialization stages using *Z. mobilis* for biochemical production. PPP: pentose phosphate pathway; G3P: glyceraldehyde 3‐phosphate; IPP: isopentenyl pyrophosphate; DMAPP: dimethylallyl pyrophosphate; GPP: geranyl pyrophosphate; FPP: farnesyl pyrophosphate; FAEE: fatty acid ethyl esters; 3‐HP: 3‐hydroxypropionic acid; 3‐HB: 3‐hydroxybutyrate; and 2,3‐BDO: 2,3‐butanediol.

Although ethanol is almost the exclusive product due to its unique physiology, *Z. mobilis* also contains endogenous metabolic pathways to produce other metabolic byproducts, such as lactate, acetate, acetoin, sorbitol and succinic acid (Fig. 1). Attempts to divert carbon flux to these products has been reported using metabolic modelling and metabolic engineering (Lee *et al*., 2010; Liu *et al*., 2010; Kim *et al*., 2014); these potential products were discussed in detail in other reviews (Rogers *et al*., 2007; He *et al*., 2014). For example, recombinant *Z. mobilis* strains transformed with *Leuconosloc* sp. D‐lactate dehydrogenase genes inserted into different genome locations associated with lactate metabolism produced D‐lactate at yields higher than 99.7% at pH 5.0 (Kim *et al*., 2014). In addition, *Z. mobilis* can also utilize sucrose to produce levan, which has properties as a cosmeceutical ingredient (Silbir *et al*., 2014), and glucose and fructose to produce gluconic acid and sorbitol (Silveira *et al*., 1999; Erzinger and Vitolo, 2006) or ethanol and sorbitol (Shene and Bravo, 2001).

Furthermore, different heterologous metabolic pathways have been engineered into *Z. mobilis* to produce chemicals for advanced biofuel or its intermediates. For example, polyhydroxylbutyrate (PHB) operon *phbCAB* was engineered into *Z. mobilis* and the enzymatic activities of PhaA and PhaB were detected with PHB accumulation (Lai and Chen, 2006). Our effort also demonstrated the possibility of engineering *Z. mobilis* for farnesene or fatty acid ethyl esters production by engineering heterologous pathway gene(s) into *Z. mobilis* (Fig. 1, unpublished data). However, the low titre of these products (e.g. 100 mg l^−1^ for farnesene) could prevent them from near term commercialization. We also explored heterologous pathways for recruiting genes from pyruvate for the production of advanced biofuels or its intermediates such as 2,3‐butanediol (2,3‐BDO, Fig. 1). Our initial effort showed that *Z. mobilis* is tolerant to 2,3‐BDO and potentially can be used for 2,3‐BDO production. We recently constructed various recombinant *Z. mobilis* strains for 2,3‐BDO production (Yang *et al*., 2016). Our results indicate that all three genes of the heterologous 2,3‐BDO biosynthesis pathway are essential for high 2,3‐BDO production in *Z. mobilis*, and our current best strain can produce 2,3‐BDO at a titre of 20 g l^−1^ in batch fermentation.

## Strain evaluation and fermentation strategies

To accelerate strain development for commercialization application, efficient and accurate high‐throughput strain evaluation and fermentation strategies have been continuously developed.

### High‐throughput strain evaluation

High‐throughput strain evaluation techniques, such as Biolog's Phenotype Microarrays, Bioscreen C, and BioLector systems have been examined. We have established phenotype profiling using the Biolog Phenotype Microarray system (Biolog, Hayward, CA, USA), which was used to profile nearly 2000 *Z. mobilis* cellular phenotypes and provided an overview of *Z. mobilis* physiology for future studies (Bochner *et al*., 2010). Several high‐throughput approaches have been widely used, including the Bioscreen C system with the capability of monitoring two 100‐well plates at 0.4 ml scale simultaneously for measuring cellular growth as discussed above (Franden *et al*., 2009, 2013; Yang *et al*., 2010b). Moreover, fermentation systems at micro‐ and mini‐scales, such as the BioLector Micro‐Bioreactor system (m2p‐Labs GmbH, Baesweiler, Germany) are advantageous for strain evaluation and culture condition optimization (Buchenauer *et al*., 2009; Funke *et al*., 2010a,b; Blomberg, 2011; Rohe *et al*., 2012; Lattermann and Büchs, 2015). BioLector Micro‐Bioreactor system has two product lines: BioLector and BioLector Pro with four robotic options (Robo S, RoboLector L, BioLector XL and RoboLector CM) and six modules for various applications (LED, FRET, anaerobic cultivation, O_2_‐upregulation, O_2_‐downregulation and CO_2_‐upregulation modules). BioLector can run 48 parallel bioreactors with online monitoring of the common fermentation parameters: biomass loading, pH, dissolved oxygen and fluorescence.

We have worked with m2p‐labs to adapt the BioLector system for *Z. mobilis* growth monitoring in different media, especially pretreated biomass hydrolysate and slurry, which is not possible by conventional absorbance‐dependent cellular growth measurements by optical density. The results showed that despite a different regression for each medium, biomass growth curves and replicates of the calibration curve were highly reproducible, and growth could be detected in the presence of hydrolysate (unpublished data). The versatile applications of this high‐throughput Micro‐Bioreactor can help accelerate research such as mutant screening, microbial physiology investigation, fermentation condition optimization and systems biology studies.

### Fermentation strategies

**Figure 2:**
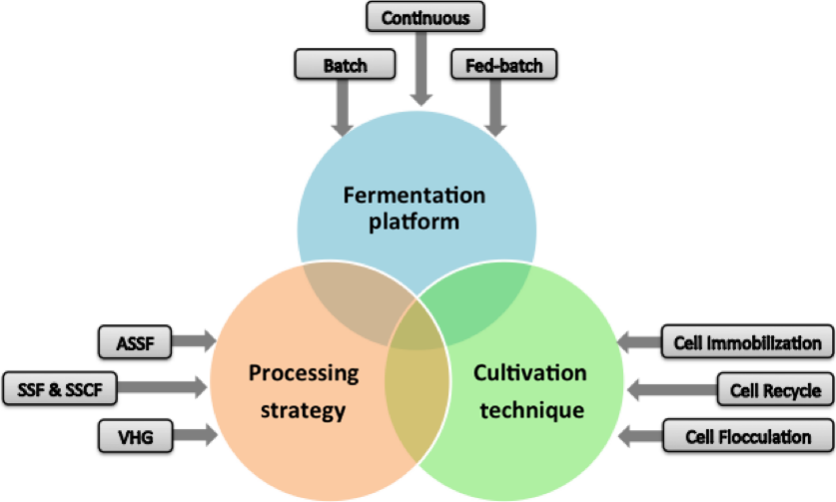
Examples of different fermentation platforms, processing strategies and cultivation techniques that have been applied on *Zymomonas mobilis*. SSF: simultaneous saccharification and fermentation; SSCF: simultaneous saccharification and co‐fermentation); ASSF: advanced solid‐state fermentation technology; VHG: very high gravity.

It will be crucial to select and optimize appropriate culture modes and strategies for maximizing product titre, yield and productivity. Different fermentation strategies have been applied for ethanol production using *Z. mobilis*, including batch, fed‐batch, continuous cultures and other fermentation techniques (Table 1, Fig. 2).

**Table 1:** Examples of different fermentation platforms, processing strategies and cultivation techniques that have been applied on *Zymomonas mobilis* for ethanol, fructose and levan production

Product	Strain	Substrate	Initial carbon (g l^−1^)	Fermentation strategy	Condition (pH, temperature, r.p.m.)	Time (h)	Titre (g l^−1^)	Reference
Ethanol	ATCC 10988	Glucose	100	Batch	pH: 4.5, 37°C	12	50.6	(King and Hossain, 1982)
	MCC 2427	Sugarcane molasses	216	Batch	pH: 5.1, 3°C	44	58.4	(Maiti *et al*., 2011)
	10225	Kitchen garbage	70	Batch	pH: 4.0, 30°C	40	52	(Ma *et al*., 2009)
	NRRL‐806	*Eucalyptus globulus*	79.5	Batch	pH: 5.5, 30°C, 150 r.p.m.	27	37	(Aroca‐Arcaya *et al*., 2014)
	CP4	Sugarcane bagasse	80	Batch‐SSF	pH: 5.0, 30°C	36	60	(dos Santos *et al*., 2010)
	8b	Paper sludge		Batch‐SSCF	pH: 5.8, 30°C, 300 r.p.m.	120	46.3	(Zhang and Lynd, 2010)
	PTCC 1718	Carob pods	180	Batch‐ASSF	pH: 5.3, 30°C	40	1.8	(Saharkhiz *et al*., 2013)
	CP4	Glucose	295	Batch‐VHG	pH: 6.0, 32°C	60	78	(Wang *et al*., 2016)
	TMY‐FHPX	Glucose	295				136	
	TMY‐FHPX	Glucose	295				145	
	TMY‐FHPX	Xylose	60				20.5	
	TMY‐FHPX	Xylose and glucose	Glucose: 170; Xylose: 60				110	
	8b	Corn stover		Cell cycle in batch, RaBIT	pH: 6.0, 30°C	24	43.4	(Sarks *et al*., 2014)
	CICC 10225	Glucose	100	Repeated batch with immobilized cells	pH: 7.0, 30°C, 140 r.p.m.	24	49.3	(Niu *et al*., 2013)
	B‐4286	Glucose	80	Fed‐batch	30°C, 150 r.p.m.	29	113	(Silman, 1984)
	WR6	Glucose	100	Continuous with flocculating cells	pH: 5.5, 30°C, 100 r.p.m.		47.6	(Fein *et al*., 1983)
	ZM4	Fructose	150	Continuous with immobilized cells	pH: 5.0, 30°C		72	(Jain *et al*., 1985)
	ZM4	Fructose	200				78.2	
Other products: Fructose (F), levan (L), 2,3‐Butanediol (2,3‐BDO) and ethanol (E)								
Fructose and ethanol	UQM 2864	Sugar cane syrup	200	Batch	pH: 5, 32°C		F: 90.5, E: 48.3	(Edye *et al*., 1989)
			350	Fed‐batch	pH: 5, 32°C	22	F: 142, E: 76.5	
Levan and ethanol	CCT 4494	Sucrose	350	Repeated batch with immobilized cells	pH: 4, 30°C, 200 r.p.m.	24	L: 21.1, E: 87.2	(Santos and Cruz, 2016)
Levan	ATCC 31821	Sucrose	250	Batch	25°C, no agitation	24	21.69	(de Oliveira *et al*., 2007)
		Sugar cane molasses	250	Batch	25°C, no agitation	24	2.53	
		Sugar cane syrup	250	Batch	25°C, no agitation	24	15.46	
	ZAG‐12	Sucrose	250	Batch	pH: 6.5, 20°C, 100 r.p.m.	72	14.67	(Melo *et al*., 2007)
	CP4	Sucrose	150	Batch	pH: 5, 25°C, no agitation	20	40.14	(Borsari *et al*., 2006)
	B‐14023	Sucrose	150	Batch	pH: 5, 28°C	48	15.52	(Silbir *et al*., 2014)
		Sucrose	299	Optimized batch	pH: 6, 28°C	42.3	40.2	
		Sucrose	299	Continuous with immobilized cells	pH: 6, 28°C	42.3	31.8	
2,3‐BDO	9C	Glucose	80	Batch	pH: 5.5, 33°C, 120 r.p.m.	24	BDO: 13.3, E: 24.9	(Yang *et al*., 2016)

SSF: simultaneous saccharification and fermentation; SSCF: simultaneous saccharification and co‐fermentation); ASSF: advanced solid‐state fermentation technology; VHG: very high gravity; RaBIT: rapid bioconversion with integrated recycle technology. 13‐H‐9‐2: 8b mutant with enhanced hydrolysate tolerance; 10225: GZNS1; CICC 10225: NRRL B‐12526; WR6: a spontaneous flocculating mutant of ATCC 29291; UQM 2864: ATCC 53431; ZAG‐12: UFPEDA 241. 9C: an 8b derivative with tetracycline and chloramphenicol resistance genes cured.

Batch cultivation in a relatively closed culture environment is the simplest mode for the production of cell mass and desired products. Because nutrients were only supplied at the beginning of fermentation and waste products (other than CO_2_) were not removed during the entire cultivation, batch cultures can only allow limited generations before growth stops. Batch fermentation mode is a good choice in the early stage of process development and research study due to its low capital investment and simple operation (King and Hossain, 1982; Veeramallu and Agrawal, 1986; Lawford *et al*., 1988; Ishikawa *et al*., 1990; Szambelan *et al*., 2004; Patle and Lal, 2007). It is also suitable for fermentation in which high cell density is not desirable. However, the drawback of the batch culture is the limitation of the low cell density and productivity. The high concentrations of toxic compounds in the fermentation broth can also influence cell growth. Therefore, fed‐batch and continuous cultures combined with flocculated or immobilized *Z. mobilis* cell systems were developed and optimized for the production of products that can reduce the inhibition effects of toxic compounds, enabling high growth rate and ethanol productivity (Arcuri, 1982; Silman, 1984; Jain *et al*., 1985; Edye *et al*., 1989; Lawford *et al*., 1998; Bravo *et al*., 2000; Amutha and Gunasekaran, 2001; Silbir *et al*., 2014). For example, the strategy of flocculating of microbial cells to increase cell densities and productivities has been widely investigated for the production of ethanol in the cultivation of *Z. mobilis* (Fein *et al*., 1983). An ethanol volumetric productivity of 80 g l^−1^ h^−1^ was attained along with an ethanol titre of 47 g l^−1^ in a continuous cultivation employing a flocculating *Z. mobilis* WR6 (Fein *et al*., 1983). A continuous culture using immobilized *Z. mobilis* cells to produce levan in Ca‐alginate gel beads was investigated, with a maximum levan titre of 31.8 g l^−1^ and productivity of 6.6 g l^−1^ h^−1^ achieved in a packed bed fermenter (Silbir *et al*., 2014). Recently, the ‘fish‐in‐net’ approach of cell immobilization has been tested with living *Z. mobilis* cells under mild conditions with mesoporous silica‐based materials as the carrier. The results showed that the encapsulated *Z. mobilis* cells did not diffuse into the surrounding medium with normal metabolism and excellent reusability (Niu *et al*., 2013). Moreover, ethanol yield from immobilized *Z. mobilis* cells, which are attached or formed a biofilm on polystyrene or delignified corn silk carriers, was higher than that from free living *Z. mobilis* using rice straw hydrolysates (Todhanakasem *et al*., 2016).

*Zymomonas mobilis* can also be used in other industrial processes for economic bioethanol production. For example, solid submerged fermentation, advanced solid‐state fermentation technology, simultaneous saccharification and fermentation, and simultaneous saccharification and co‐fermentation have been used for ethanol production (Lawford *et al*., 1997; Zhang and Lynd, 2010; Das *et al*., 2013; Saharkhiz *et al*., 2013). VHG fermentation is the mainstream technology in the ethanol industry with fermenting medium containing sugar more than 250 g l^−1^, and recombinant *Z. mobilis* strain TMY‐HFPX can produce ethanol up to 136 g l^−1^ from 295 g l^−1^ glucose with a theoretical yield of 90% in VHG fermentation (Wang *et al*., 2016). However, if biomass feedstocks are used, high solid loading will increase the concentration of lignocellulose‐derived inhibitors and also the osmolarity. Together with high concentrations of end‐product (ethanol), it will be very challenging for *Z. mobilis* to keep its robust fermentation performance, especially in the xylose utilization stage. We found that by increasing the inoculation could improve the tolerance of *Z. mobilis* to lignocellulosic hydrolysates. In addition, the Rapid Bioconversion with Integrated recycle Technology process was developed to reduce capital costs, processing times, and biocatalyst costs and *Z. mobilis* performed well in this fermentation process among nine recombinant microbial strains tested, including model microbial biocatalysts of yeast and *E. coli* (Sarks *et al*., 2014).

## Classical genetics tools and emerging technology

A significant collection of classical genetics tools have been explored and are now routine metabolic engineering practices in *Z. mobilis*, including stable and transferable plasmids, shuttle vectors, promoters, transformation methods such as conjugation and electroporation, reporter genes such as green fluorescent protein (GFP) and ice nucleation activity, and transposon mutagenesis strategies (Skotnicki *et al*., 1980; Carey *et al*., 1983; Browne *et al*., 1984; Conway *et al*., 1987a,b; Arfman *et al*., 1992; Delgado *et al*., 1995; Drainas *et al*., 1995; Zhang *et al*., 1995, 2013a; Pappas *et al*., 1997; Douka *et al*., 2001; Yang *et al*., 2010a,b, 2015; Dong *et al*., 2011, 2013; Pappas, 2011; Jia *et al*., 2013; Dunn and Rao, 2015; Yi *et al*., 2015; Wang *et al*., 2016). These methods have been widely reviewed (Panesar *et al*., 2006; He *et al*., 2014) and are not further described here.

Nevertheless, it is worthwhile to mention that the investigation of DNA restriction‐modification (R‐M) systems in *Z. mobilis* helps improve transformation efficiency for more amenable strain development (Kerr *et al*., 2011; Wu *et al*., 2013). Inactivation of the type IV R‐M element ZMO0028 resulted in 60‐fold increase when unmethylated plasmid DNA was used. Furthermore, transformation efficiencies increased 30‐fold in a mutant strain of putative type I DNA methyltransferase S subunit (ZMO1933) when methylated plasmid DNA was introduced (Kerr *et al*., 2011). A similar result was reported by an independent study, where the inactivation of ZM00028 and ZM01933 significantly improved electroporation efficiency (by 17‐fold and twofold respectively) when methylated plasmid DNA was used (Wu *et al*., 2013). In addition, the major physiological traits of growth, glucose utilization and ethanol yield have not been significantly changed in these R‐M mutants, although the ZMO0028 mutant was reported to have an increased maximum specific growth rate and biomass yield in one study (Kerr *et al*., 2011).

### Systems biology‐based strategies

Recent rapid progress in such techniques as next‐generation sequencing and systems biology have been extensively applied to metabolic engineering (Tyo *et al*., 2010). Indeed, genome sequencing projects provide opportunities for fundamental insights to facilitate strain development (Jeffries, 2005). Moreover, the recent and continuous breakthroughs in systems biology and sequencing technologies have changed the paradigm strategies for industrial biocatalyst development (Atsumi *et al*., 2008, 2009, 2010; Kim *et al*., 2008; Prather and Martin, 2008; Connor and Liao, 2009; Lee, 2009; Picataggio, 2009; McArthur and Fong, 2010; Na *et al*., 2010; Tyo *et al*., 2010; Yang *et al*., 2010b; Brown *et al*., 2011) and help us understand the biocatalysts at a global level for future systematic functional redesign (Park *et al*., 2008; Yang *et al*., 2009b, 2010a,b). Detailed description of systems biology studies in *Z. mobilis* can be found in a recent review (He *et al*., 2014).

**Figure 3:**
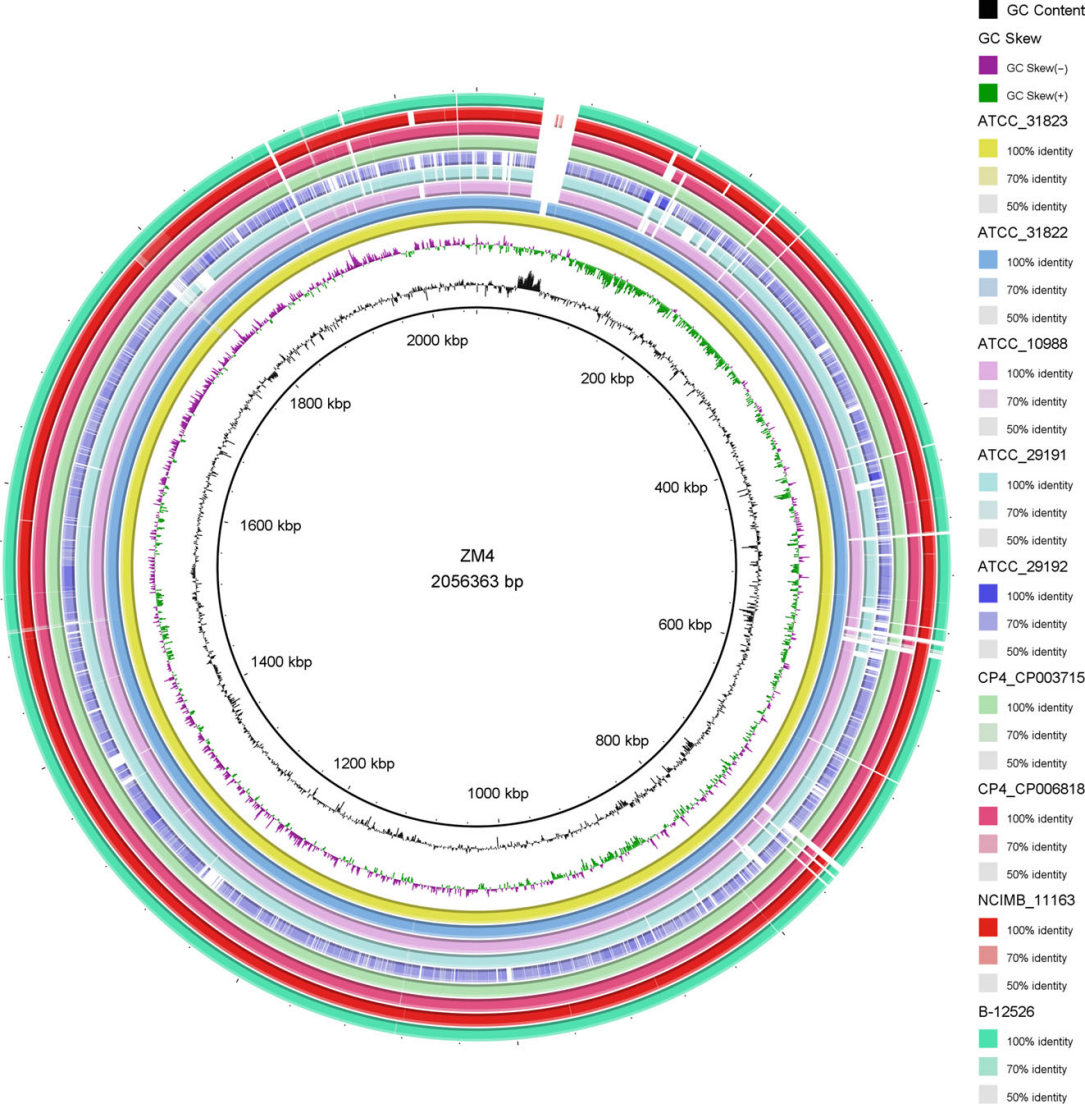
*Zymomonas mobilis* comparative genomic analysis. The BLAST Ring Image Generator (BRIG) software was used to compare *Z. mobilis* strains whose genome sequences were available at NCBI database. Genome sequence of different strains, indicated by different coloured concentric circles, are as follows from outermost circle to inward: B‐12526 (NZ_CP003709.1), NCIMB 11163 (NC_013355.1), CP4_CP006818 (NC_022900.1), CP003715 (NZ_CP003715.1), ATCC 29192 (NC_015709.1), ATCC 29191 (NC_018145.1), ATCC 10988 (NC_017262.1), ATCC 31822 (GCA_000303025.1), ATCC 31823 (GCA_001563365.1), GC skew (+, purple; ‐, green) and GC content. All *Z. mobilis* genomes were compared to strain ZM4 (NC_006526.2).

The *Z. mobilis* model strain, ZM4, has a small genome size (ca. 2 Mb, Seo *et al*., 2005). The genome annotation has been improved recently (Yang *et al*., 2009b), which greatly assists in the accumulation of *Z. mobilis* systems biology data, especially the microarray‐based transcriptomic datasets of different strains grown under different conditions (Yang *et al*., 2009a, 2010b, 2013, 2014a,b; Hayashi *et al*., 2012; He *et al*., 2012a,b; Jeon *et al*., 2012; Skerker *et al*., 2013; Yi *et al*., 2015; Zhang *et al*., 2015). The availability of several other *Z. mobilis* genomes, such as CP4, NCIMB 11163, ATCC 29191, ATCC 29192, ATCC 10988 and ZM4 mutants of ATCC 31822 and ATCC 31823 (Kouvelis *et al*., 2009, 2011, 2011, 2014; Peralta‐Yahya and Keasling, 2010; Pappas *et al*., 2011; Smith and Liao, 2011; Desiniotis *et al*., 2012; Zhao *et al*., 2012, 2016) and other strains in the sequencing pipeline makes comparative genomics research practical. For example, we used the model strain, ZM4, as a reference to compare nine other strains with genome sequences using the Blast based Ring Image Generator (Alikhan *et al*., 2011). The result demonstrated that these strains are closely related; displaying a high degree of similarity, both through synteny and homology, at the genome level (Fig. 3). A noticeable difference among these strains was the presence of a 25 kb unique region in strain ZM4, that is, absent from seven strains, but with partial coverage in ATCC 31823 and ATCC 31822. This unique region in strain ZM4 spans 36 open reading frames (ORFs, ZMO1930‐ZMO1971) with most genes encoding hypothetical proteins, phage related integrase family protein, and Type IV secretory pathway protease. It is possible that this region was obtained through horizontal gene transfer, although further confirmation is necessary. The uniqueness of this region to strain ZM4 was further supported by OrthoMCL based clustering analysis, where most of the genes within this region were clustered only with two taxa (ATCC 31823 and ATCC 31822). These results are also supported by the genomic blast based dendrogram result available on the NCBI website suggesting that strains ATCC 31823 and ATCC 31822 share the highest synteny and homology with strain ZM4. The uniqueness of this region is also consistent with the genetic background of these strains, since ATCC 31822 is a flocculating mutant of ZM4 (Zhao *et al*., 2012), and ZM481 (ATCC 31823) is an ethanol‐tolerant strain derived from ZM4 (Zhao *et al*., 2016). Differences between ZM481 and ZM4 have been analysed and results show that except for 146 single‐nucleotide polymorphisms (SNPs), ZM481 and ZM4 are almost same with no insertions–deletions (indels) identified, suggesting that SNPs may be responsive for the ethanol tolerance of ZM481 mutant (Zhao *et al*., 2016).

Metabolic engineering uses DNA technology to modify the direction of metabolic fluxes towards a desired product. However, the incorporation of foreign gene/pathways or stressful conditions often switches cellular metabolism or redox balance to alternative pathways, which can lead to the accumulation of toxic or unwanted intermediates, thereby decreasing the overall yield of the system. The traditional engineering approach to overcome these problems is to design a computational model (i.e. a network) of metabolism that is based on available omics and kinetic data. Various metabolic flux (network) models have been developed to guide strain development. The primary method has been metabolic flux analysis (Moreno‐Sánchez *et al*., 2008; Lee *et al*., 2011), in which fluxes are determined in an attempt to identify limiting steps. Flux balance analysis, a stoichiometry constraint‐based approach for estimating fluxes under different conditions (Raman and Chandra, 2009), is an example of these tools. A number of metabolic network models have been developed for *Z. mobilis* (Altintas *et al*., 2006; Lee *et al*., 2010; Widiastuti *et al*., 2011; Pentjuss *et al*., 2013; Rutkis *et al*., 2013; Kalnenieks *et al*., 2014). Among these, three medium‐scale and two genome‐scale stoichiometric metabolic network models have been reported. Recently, a simulation‐ready model of the ED pathway of *Z. mobilis* (comprising only 16 enzymatic reactions) was built (Pentjuss *et al*., 2013; Rutkis *et al*., 2013; Kalnenieks *et al*., 2014). Metabolic control analysis of this model pointed to ATP turnover as a major bottleneck, suggesting that to increase the glycolytic flux in *Z. mobilis*, single enzymes of the ED pathways should not be considered as a prime target for overexpression.

Systems biology makes use of these computational models to try to understand perturbative metabolic effects. In addition, we reported a paradigm which combines classical genetic methods and systems biology tools to unravel tolerance mechanisms for toxic components derived from cellulosic hydrolysates. Using this approach, individual inhibitor tolerance genes were identified in *Z. mobilis*, providing promise for strain improvement (Yang *et al*., 2010a,b, 2012). The availability of systems biology datasets; as well as large‐scale phenotypic datasets (e.g. 492 datasets in different growth conditions) obtained by investigating the phenotype of a barcoded mutant library (Skerker *et al*., 2013; Kosina *et al*., 2016); will further facilitate genome‐scale metabolic modelling to understand microbial physiology and to guide metabolic engineering efforts (Lee *et al*., 2010; Widiastuti *et al*., 2011; Motamedian *et al*., 2016).

### Synthetic biology‐based pathway engineering

Genes from *Z. mobilis* contribute to the standard biology of the International Genetically Engineered Machine registry, especially with unique characteristics such as *pdc* (ZMO1360, EC: 4.1.1.1), *adhB* (ZMO1596, EC:1.1.1.1), extracellular sucrase gene *sacC* (ZMO0375, EC:3.2.1.26), and *glf* (ZMO0366, glucose facilitated diffusion protein). For example, the ethanol production module of the fusion enzymes, Pdc and AdhB from *Z. mobilis* (Part:BBa_K1122673), could increase ethanol yields and productivity in *E. coli* and lactic acid bacteria (Nichols *et al*., 2003; Chen *et al*., 2009; Flynn *et al*., 2010; Lewicka *et al*., 2014).

To accelerate metabolic engineering practices, we have recently constructed a 3.0‐Kb Biobrick‐compatible minimized shuttle vector for efficient pathway construction with the potential of maximum pathway gene size. This shuttle vector contains only the essential elements of origins of replication for both *E. coli* and *Z. mobilis*, an antibiotic marker of the spectinomycin resistance gene *addA*, multiple cloning sites and Biobricks adapters (Yang *et al*., 2016). We also identified various promoters with different strengths based on systems biology data and verified good correlation using GFP reporter gene experimentally. In addition, two inducible promoters have also been tested and confirmed to work in *Z. mobilis*. These were applied to the 2,3‐BDO heterologous pathway engineering work (Yang *et al*., 2016).

Recently, the strategy of global transcription machinery engineering has also been applied in *Z. mobilis* to improve furfural and ethanol tolerance by constructing and screening the random mutagenesis libraries of sigma factor RpoD obtained through error‐prone PCR. This approach will be effective for improving other similar complex phenotypes involved in multiple genes going forward (Tan *et al*., 2015, 2016).

In addition, work to understand the mechanism of other genetic factors impacting gene regulation is ongoing, which potentially could be applied to synthetic biology applications,. For example, the presence and potential role of small RNAs (sRNAs) in *Z. mobilis* was investigated by computational prediction and molecular biology experimental approaches. Fifteen novel sRNAs were confirmed with three sRNAs (Zms2, Zms6 and Zms18) differentially expressed under ethanol stress (Cho *et al*., 2014). This result suggests the regulatory role of sRNAs in ethanol production or tolerance in *Z. mobilis*, as well as the potential application of RNA‐associated mechanisms for metabolic engineering practice.

Finally, CRISPR‐Cas systems in *Z. mobilis* has been investigated recently and the results showed that *Z. mobilis* type I‐F CRISPR‐Cas system was expressed and active in immune interference under normal growth conditions (Dong *et al*., 2016). Dunn reported in her thesis that a type II CRISPR/Cas expression system was constructed in *Z. mobilis* and that small RNAs can direct the Cas9 nuclease to target the *Z. mobilis* genome for genome editing (Dunn, 2015). These findings suggest that the existence of endogenous type I‐F CRISPR‐Cas system in *Z. mobilis* will not affect the exogenous type II CRISPR/Cas expression system for genome engineering, such as editing, interfering, tagging, screening and visualizing (Sander and Joung, 2014). In addition, a DNA‐guided nuclease *Natronobacterium gregoryi* Argonaute (NgAgo) was reported recently, which can introduce targeted double strand breaks, and is suitable for genome editing (Gao *et al*., 2016). Compared with the CRISPR/Cas9 technique, the NgAgo system could be a very exciting genome‐editing technique, which uses DNA instead of RNA as the guide without the requirement of a protospacer adjacent motif site. Moreover, several features could indicate higher fidelity of this system relative to that of CRISPR‐Cas systems including the longer DNA guide (24 nucleotides versus 20 nucleotides) for Cas9 gRNA, higher sensitivity to single base mismatches, and better performance on GC‐rich regions (Gao *et al*., 2016). Although only mammalian cells were used in this study, it can be expected that further studies could demonstrate its applicability on prokaryotic systems like *Z. mobilis* in the future.

## Perspectives

In summary, *Z. mobilis* can serve as a model for biofuel and biochemical production and the knowledge gained from model strain studies can be extended towards the development of additional biocatalysts. For example, previous studies provided evidence that the overexpression of a sodium proton anti‐porter gene *nhaA* and a global regulator *hfq* in *Z. mobilis* elevated its tolerance to sodium acetate, and overexpression of the homologous genes in yeast helped that organism resist sodium acetate (Yang *et al*., 2010a,b). Another example is the optimized isobutanol pathway established in *E. coli* (Atsumi *et al*., 2008, 2009, 2010; Connor and Liao, 2009), which has been applied for isobutanol production in yeast by Gevo (Englewood, CO, USA).

However, several questions have not been completely resolved and challenges still need to be overcome. First of all, it is unexpected that the facultative anaerobic *Z. mobilis* has the ED pathway, which is usually associated with obligate aerobic microorganisms. In addition, *Z. mobilis* has a unique energy‐uncoupled growth. This unique carbon and energy metabolism therefore confers to *Z. mobilis* the very desirable characteristic of high ethanol production and tolerance. However, there have been no systematic investigations of the relationships between carbon metabolism, energy metabolism and environmental factors. There have also been limited studies on the impact of carbon metabolism on ethanol production, although a recent paper investigated the interaction between respiration and glucose catabolism and indicated that respiration accelerates glucose consumption in non‐growing cells of *Z. mobilis* (Rutkis *et al*., 2016). Furthermore, several attempts have been reported to delete the *pdc* gene without success, suggesting the essential nature of the *pdc* gene. Although it is advantageous for *Z. mobilis* to have the unique *pdc* and *adh* genes for efficient ethanol production, the essential characteristics of the *pdc* gene makes the carbon diversion from ethanol production to other desired products very challenging, and should be addressed.

In conclusion, although *Z. mobilis* is amenable to metabolic engineering with various genetics tools available already, more sophisticated and efficient tools for genome editing are still needed, such as the CRISPR/Cas9‐based genome‐editing tools for genome modification.

## Conflict of Interest

None declared.
